# *In silico* and *Ex vivo* approaches identify a role for toll-like receptor 4 in colorectal cancer

**DOI:** 10.1186/1756-9966-33-45

**Published:** 2014-05-22

**Authors:** Daniel A Sussman, Rebeca Santaolalla, Pablo A Bejarano, Monica T Garcia-Buitrago, Maria T Perez, Maria T Abreu, Jennifer Clarke

**Affiliations:** 1 Division of Gastroenterology, Department of Medicine, University of Miami, 1120 NW 14th Street, Clinical Research Building 310J, Miami, FL 33136, USA; 2 Department of Pathology, Cleveland Clinic, Weston, 2950 Cleveland Clinic Boulevard, Weston, FL 33331, USA; 3 Department of Pathology, University of Miami, 1120 NW 14th Street, Clinical Research Building, 14th Floor, Miami, FL 33136, USA; 4 Department of Pathology, JFK Medical Center, 5301 S Congress Ave JFK Pathology Dept, Atlantis, FL 33462, USA; 5 Food Science and Technology Department, University of Nebraska-Lincoln, 322 Food Industry Complex, Lincoln, NE 68583-0919, USA

**Keywords:** Colon cancer, Colorectal cancer, TLR4, Bioinformatics, Transcriptome, Immunohistochemistry

## Abstract

**Background:**

Inflammation increases the risk of colorectal cancer (CRC). We and others have described a role for TLR4, the receptor for LPS, in colon cancer. To explore the relationships between TLR4 expression and CRC, we combined the strength of transcriptome array data and immunohistochemical (IHC) staining.

**Methods:**

TLR4 signal intensity was scored in the stromal and epithelial compartments. Detection of differential expression between conditions of interest was performed using linear models, Cox proportional hazards models, and empirical Bayes methods.

**Results:**

A strong association between TLR4 expression and survival was noted, though a dichotomous relationship between survival and specific TLR4 transcripts was observed. Increasing TLR4 expression was seen with advancing tumor stage and was also over-expressed in some adenomas. IHC staining confirmed the positive relationship between TLR4 staining score in the CRC tumor stroma and epithelium with tumor stage, with up to 47% of colon cancer stroma positive for TLR4 staining. Increased TLR4 expression by IHC was also marginally associated with decreased survival. We now also describe that pericryptal myofibroblasts are responsible for a portion of the TLR4 stromal staining.

**Conclusions:**

Increased TLR4 expression occurs early in colonic neoplasia. TLR4 is associated with the important cancer-related outcomes of survival and stage.

## Introduction

A growing body of evidence supports the notion that inflammation and colorectal cancer (CRC) are interrelated, including clinical observations and animal models [1]. The colonic mucosa is in constant contact with a high density of diverse microorganisms [2]. Antigens from these microbes are recognized by pattern-recognition receptors of the innate immune system. The toll-like receptor (TLR) family represents a critical part of this innate immune recognition, with each TLR recognizing pathogen-associated- or damage-associated-molecular patterns (DAMPs) [3]. In particular, TLR4 recognizes lipopolysaccharide (LPS) from the outer membrane of Gram-negative bacteria, the most common type of colonic bacteria [4]. Moreover, TLR4 is a receptor for DAMPs like hyaluronic acid and S100A9 [5,6]. Our laboratory has studied the role of TLR4 in intestinal inflammation and colitis-associated neoplasia, supporting the function of TLR4 as a tumor promoter in human tissue and murine models [7,8]. Our work in sporadic CRCs also links TLR4 to activation of the neoplastic Wnt/β-catenin pathway [9].

In this study, we wished to characterize the role of TLR4 in the natural history of sporadic colonic neoplasia. The objective was to identify the prevalence of altered TLR4 RNA expression and tissue localization in sporadic neoplasia, and to determine the relationship between TLR4 expression and survival in CRC. We combined the strengths of transcriptomic array data and immunohistochemical (IHC) staining. Analysis of arrayed data offers a method by which to efficiently query the genomic and protein expression within a given tissue offering insight into the influence of gene expression on patient phenotypes. In an effort to establish the influence of TLR4 on CRC behavior, we drew upon genomic data sets and validated RNA expression profiles with immunofluorescent (IF) staining for theTLR4 protein from tissue microarrays (TMAs) obtained from the National Cancer Institute (NCI).

Our results demonstrate that TLR4 is expressed in adenomas and CRCs. Up to 47% of sporadic colon cancers express TLR4 protein with meaningful impact on survival and other clinical indices. Expression in tumors is localized predominantly in the stromal compartment, with a notable increase in pericryptal fibroblasts in the lamina propria.

## Methods

### Gene expression profiling

Gene expression arrays were identified by search of the Gene Expression Omnibus (GEO) database [10]. Data sets were searched using the terms “colon cancer”, “colorectal cancer”, “rectal cancer”, “colon polyp”, and “colorectal neoplasia”. Searches were limited to expression data (messenger RNA). Data sets were included if they contained paired human samples ≥16 subjects, included accompanying clinical data, and had annotation files indicating TLR4. Studies were excluded if they used only animal or cell line models.

Keyword search (November 2011) revealed 170 CRC data sets. 97 pertained to human CRC, and 64 consisted of greater than or equal to 16 samples. 29 contained information on TLR4 expression with clinical characteristics of interest, including demographics, histologic progression of dysplasia, polyp size, histology, initial CRC stage, tumor grade, metastasis, survival (overall [OS], disease specific [DSS], disease free [DFS]), recurrence, and microsatellite instability (MSI).We then reorganized data by pairs of probes to observe the influence of varying transcript length on outcomes. Eleven studies were ultimately selected. A second GEO search was performed to identify series that stratified expression data by tissue compartment (ie, epithelium vs stroma) to further clarify TLR4 localization.

### Tissue microarrays

TMA slides were provided by the NCI Cancer Diagnosis Program (CDP). Other investigators may have received slides from these same array blocks. The CDP arranged 279 colon tissue specimens with 182 CRCs of mixed stages and matched normal tissues on two slides [11]. Neoplastic tissue consisted of adenomas (n = 19) and American Joint Committee on Cancer (AJCC) CRC stage I (n = 24), II (n = 61), III (n = 72), and IV (n = 25). Diverticulitis samples served as inflammatory, non-cancer controls. De-identified clinical data were provided by the CDP. Additional polyps with normal controls were stained on proprietary TMAs (US Biomax).

### IF scoring

IF staining was performed on TMAs to detect human TLR4 (Novus Biologicals). Pan-cytokeratin was used as a counterstain to highlight intestinal epithelium (Abcam), and DAPI to counterstain nuclei. TLR4 detection was enhanced using conjugated Tyramide with the fluorochrome Alexa Fluor 488 (Invitrogen). Pan-cytokeratin was detected using an anti-rabbit secondary antibody conjugated with Alexa Fluor 647 (Invitrogen). Stained slides were scanned (Olympus VS120) and viewed using OlyVIA 2.4. A Leica TCS-SP5 Confocal was used for triple IF images. Staining patterns, intensity quantification, and extent TLR4 by surface area were determined by two senior GI pathologists (PAB and MTG) masked to diagnoses. A training subset was independently interpreted and inter-observer variation was determined. Moderate agreement was noted for the stromal score (weighted κ = 0.58 [95%CI 0.28-0.89]); moderate-to-strong agreement was observed for epithelium (weighted κ = 0.68 [95%CI 0.39-0.97]). Disagreement between scoring was settled by consensus. TLR4 signal intensity was scored in the stroma and epithelium. The signal intensity was scored as 0, no TLR4 staining; 1+, low intensity; 2+, moderate intensity; or 3+, high intensity. The extent of surface area with TLR4 was scored on a scale of 0–3 (0: no staining; 1+: present, but <20%; 2+: 20–50%; and 3+: >50%). A TLR4 positivity score was calculated by multiplying staining intensity and surface area data by tissue compartment (range: 0–9) [7,12,13].

To qualify TMA observations, IHC was performed on normal colon, adenomas, and CRCs for TLR4 (Novus Biologicals), smooth muscle actin (α-SMA, Abcam), vimentin (Cell Signaling), and CD68 (Dako) on curls from tissue blocks. Secondary antibody conjugated with horseradish peroxidase was used prior to incubation with the substrate 3,3′-diaminobenzidine. Samples were counterstained with hematoxylin and scored (pathologist MTP).

Approval by the university’s Institutional Review Board was obtained.

### Data analysis

#### Gene expression data

Analysis included quality control assessments of processed data. Differential expression discovery was performed using linear models and empirical Bayes methods (t-tests and ANOVA) via R statistical language [14]. Survival analyses were conducted using Cox proportional hazards, with results corrected for multiple comparisons using false discovery rate procedures [15]. Results were assessed for biological relevance. Where processed data were unavailable, raw data were analyzed with Robust Multichip Average using background adjustment, quantile normalization, and summarization; R language was used for Affymetrix chips and compared to processed samples. p-values <0.1 were considered significant. The p-value cut-off of 0.1 was selected as this value represents a favorable compromise between false positive and true positive rates in the setting of background “noise” associated with the identification of differentially expressed candidate RNAs with microarray data [16].

#### Tissue microarray data

TLR4 staining intensity, surface area, and intensity score were correlated with clinico-pathologic endpoints. An arbitrary TLR4 intensity score of >3 was selected to denote positive TLR4 staining, with a score of >5 considered strongly positive. R software was used to reveal relationships according to guidance provided by the CDP [11]. Non-parametric Wilcoxon sum-rank tests were performed for non-normal distributions.

## Results

### Gene expression data

**Figure 1:**
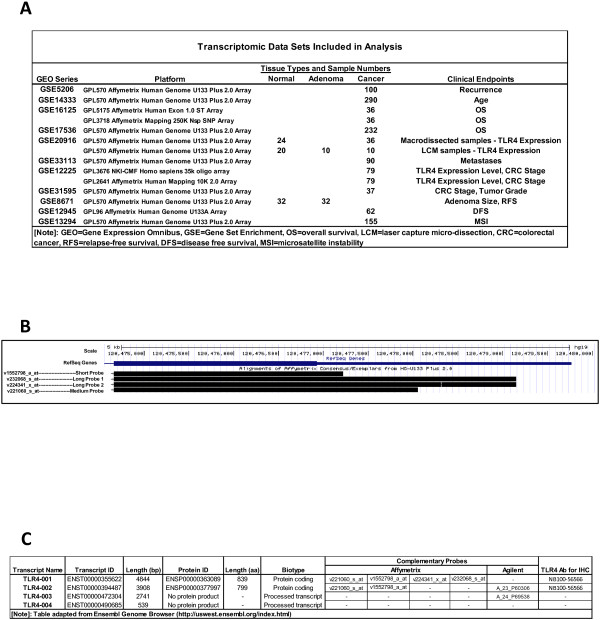
**Data Sets and Description of Probes with Corresponding Transcripts. A)** Transcriptome data sets included in analysis with GSE Series Number as identified on GEO. Platform used, colon tissue type studied, numbers of tissues included, and clinical endpoints are listed. **B)** TLR4 Gene and Transcripts. Assembly of known TLR4 gene and mRNA transcripts using University of California at Santa Clara Genome Browser. The size of the transcript identified by the individual Affymetrix probes varies and we have denoted them as follows: v1552798aat (Short Probe), v232068sat (Long Probe 1), v224341xat (Long Probe 2), and v221060sat (Medium Probe). **C)** TLR4 Transcript Table. Description of known transcript variants by length of sequence and protein products where applicable. Complementary probes by platform manufacturer and antibodies for IHC are detailed. This table was adapted from Ensembl Genome Browser.

11 data sets met our strict entry criteria (Figure 1A).The most commonly included platform was an Affymetrix chip employing four distinct TLR4 probes (Figure 1B). For ease, we have relabeled these probes by transcript length: v1552798 = Short, v221060 = Medium, v232068 = Long1, and v224341 = Long2 (Figure 1C).

#### Demographics and colonic tumor location

Meaningful data regarding patient age at time of CRC diagnosis was available in four studies (GSE14333, GSE16125, GSE33113, and GSE31595). In one series, increasing age was associated with higher TLR4 expression, but the effect was minor with a regression coefficient (coef) = 1.02 (p = 0.018) (GSE14333) [17]. In the remaining studies, no consistent relationship between age, gender, ethnicity, colonic location, and TLR4 expression was noted. No relationship between TLR4 and adenoma size was identified (GSE8671) [18].

#### TLR4 expression is increased in colon adenomas and CRC

In an effort to clarify the temporal relationship between TLR4 expression and colonic neoplasia, we identified data sets reporting normal tissue, adenomatous polyps, and CRC. Skrzypczak, et al. examined surgical specimens from 105 patients comparing CRC to matched normal tissue. All four TLR4 probes were significantly different between carcinoma and matched normals, with lower median expression observed in normal tissue (Normal vs Carcinoma: Short 3.48 vs 4.63, p = 4.16 × 10^−7^; Medium, 3.25 vs 4.78, p = 4.97 × 10^−5^; Long1, 4.66 vs 6.58, p = 3.22 × 10^−8^; Long2, 5.63 vs 7.07, p = 8.61 × 10^−9^)(GSE20916) [19].

**Figure 2:**
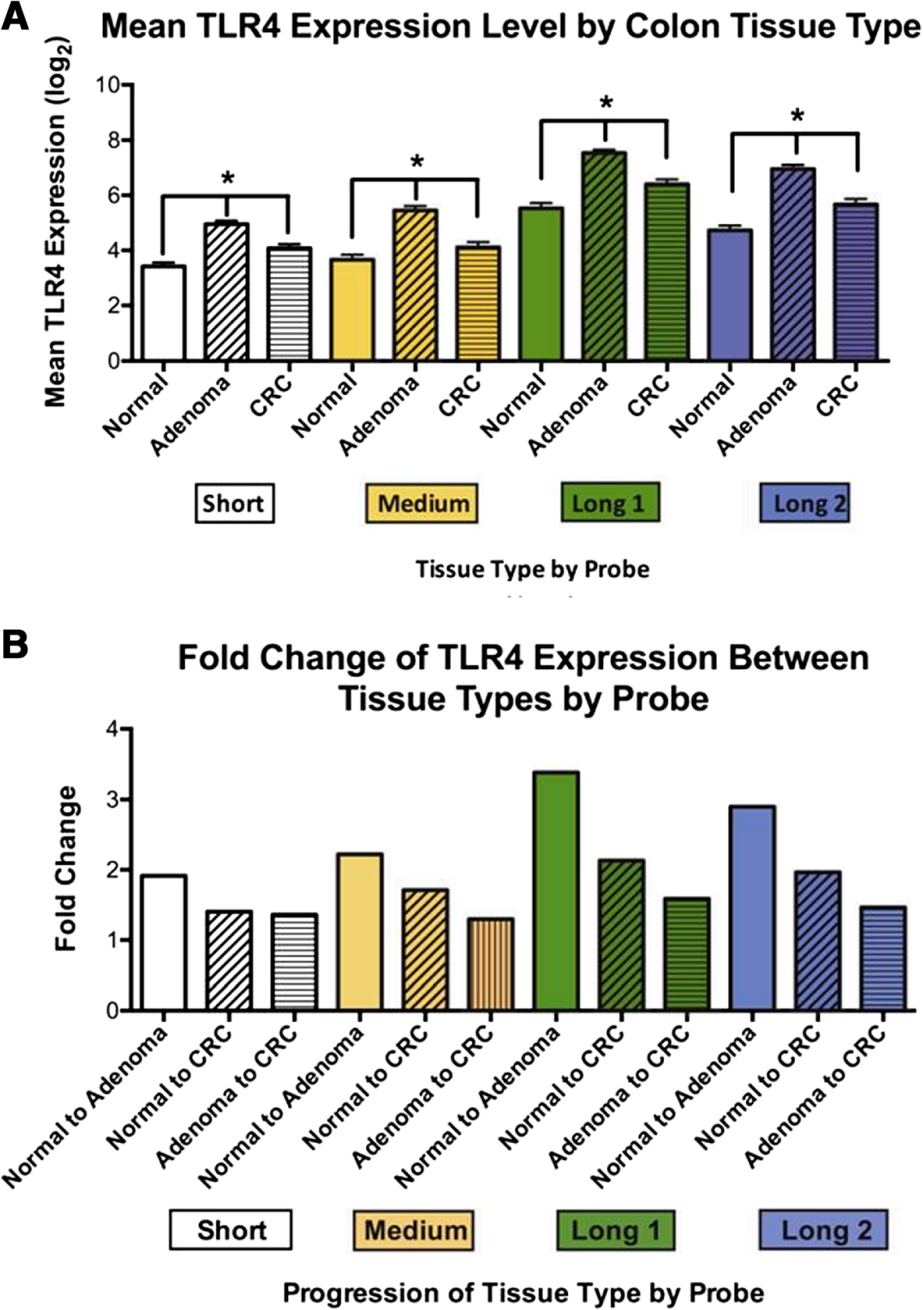
**TLR4 Expression by Colon Tissue Type. A)** Mean TLR4 expression for normal colon, adenoma, and CRC stratified by each of the 4 probes for TLR4. Mean TLR4 expression was higher in colonic neoplasia than normal tissue for all probes with the macro-dissected specimens from GSE20916. **B)** Fold change for TLR4 expression was calculated using RMA. Mean FC for the normal-to-CRC, normal-to-adenoma, and adenoma-to-cancer samples for each TLR4 probe are presented. The lowest grade of histology is the reference standard for comparison within each column. The highest TLR4 fold change (FC) is in adenoma-compared-to-normal among all tissues tested.

We then asked whether TLR4 expression is increased in the important adenocarcinoma precursor, adenomatous polyps. All four probes for TLR4 were significantly different between normal tissue and adenomas or cancer (Figure 2A). TLR4 expression was higher in adenomas than cancers; length of TLR4 transcript had no influence. This observation was confirmed in a separate series considering all CRC stages in aggregate (GSE12225) [20]. This series found that malignant neoplastic tissue had lower TLR4 expression than adenomas from patients with CRCs (adenoma vs malignancy: 0.54 vs 0.06, coef = −0.43, p = 0.021) (GSE12225). This relationship held true among all colon cancer stages. Tumor fractions consisting of a mixture of adenoma and carcinoma, earlier stages of cancer, and carcinomas with lymph node metastasis, all had lower TLR4 expression than adenomas with low-grade dysplasia (coef = −1.81, p = 0.043; coef = −1.56, p = 0.058; and coef = −1.27, p = 0.05, respectively) (GSE12225). RMA expression analysis was performed to show fold change (FC) for TLR4 expression between tissue types. TLR4 FC increase was highest for adenoma-compared-to-normal (mean FC in Figure 2B). The data demonstrate that TLR4 expression is at least doubled in adenomas and colon cancers compared with normal tissue.

#### TLR4 expression shifts to the stromal compartment in CRC

One of the shortcomings of arrayed tissues is that RNA expression data are derived from a composite of epithelial cells and the surrounding stroma. For CRC, this distinction is important to discern whether the tumor-promoting signal comes from the malignantly transformed epithelial cells or the surrounding stromal components. One data set in GEO consisting of 13 CRCs and 4 matched normal tissues separated tissue into epithelial and stromal compartments by laser capture microdissection (GSE35602) [21]. TLR4 expression was higher in the stromal tissue than malignant epithelium of CRC (coef = 1.21, p = 0.077). Matched normal tissues derived from colon remote from tumor showed trends toward lower TLR4 expression in CRC epithelium compared to normal epithelium (coef = 0.68, p = 0.18). Among normal tissues, TLR4 expression was similar in the stroma and epithelium, while in tumors expression was higher in the stroma relative to epithelium, i.e., the relative expression of stromal TLR4:epithelial TLR4 is higher in malignant tissue than matched normals.

#### TLR4 expression is associated with CRC stage

We next sought to determine the relationship between TLR4 expression and CRC stage. It is often difficult to predict which patients with stage II and stage III colon cancer will benefit from chemotherapy [22,23]. Thorsteinsson, et al. studied 37 patients with stage II and III colon cancer; TLR4 expression was significantly higher in stage III tumors than stage II for two of the four TLR4 probes (Medium, p = 0.061 and Long2, p = 0.092) (GSE31595) [24]. TLR4 expression was numerically, but not statistically, higher in stage III tumors for the remaining probes (Short, p = 0.466 and Long1, p = 0.117).

By contrast, advanced rectal cancer with nodal metastases has decreased TLR4 expression compared with earlier stage rectal cancer (coef = −0.44, p = 0.079) (Table 1) (GSE12225) [20]. This relationship also held true when comparing subjects with nodal metastases or advanced local disease, T3N0, with node-negative, early stage rectal cancer (coef = −0.53, p = 0.029) (GSE12225).

**Table 1:** TLR4 expression and tumor stage

**Rectal cancer - GSE12225**			
**Experimental group**	**Control**	**Coef**	**p-value**
**Adenocarcinoma**	**Adenoma**		
**AC + CA + CC + CC(N)**	**AA**	**−0.4333**	0.0208*
**T2 stage with nodal metastases**	**No nodal Metastases**		
**T2N1 + T2N2 + T2N3**	**T0N0 + T1N0 + T2N0 + T3N0 + TisN0**	**−0.442**	0.0787*
**T2 stage with nodes and T3 stage without nodes**	**Lower stage without nodes**		
**T2N1 + T2N2 + T2N3 + T3N0**	**TisN0 + T0N0 + T1N0 + T2N0**	**−0.529**	0.0289*
**Stage III relative to stage II - GSE31595**			
**Probe**	**Coef**	**p-value**	
**Short probe**	**0.105**	**0.466**	
**Medium probe**	**0.43**	0.061*	
**Long probe 1**	**0.744**	**0.117**	
**Long probe 2**	**0.695**	0.092*	

Notes:

[1] Coef = regression coefficient, AA = Adenoma, AC = Adenoma fraction from cases with a carcinoma focus, CA, tumor fractions consisting of a mixture of adenoma and carcinoma tissue, CC = carcinomas without lymph node metastasis, CC (N) = carcinomas with lymph node metastasis, TxNx = tumor size/extension and nodal status as part of the TNM staging system, * = statistically significant.

TLR4 expression is significantly lower in later stage than earlier stage rectal cancer (coef < 0 signifies a negative relationship of the experimental compared to control group, while coef > 0 signifies a positive relationship of the experimental compared to control group). Subjects having nodal metastases express lower TLR4 than those without (GSE12225). In a separate series of patients with stage II and III colon cancer, TLR4 expression was higher in stage III tumors than stage II for two of the four TLR4 probes (Medium Probe and Long Probe 2) (GSE31595).

#### TLR4 expression is strongly associated with survival

**Figure 3:**
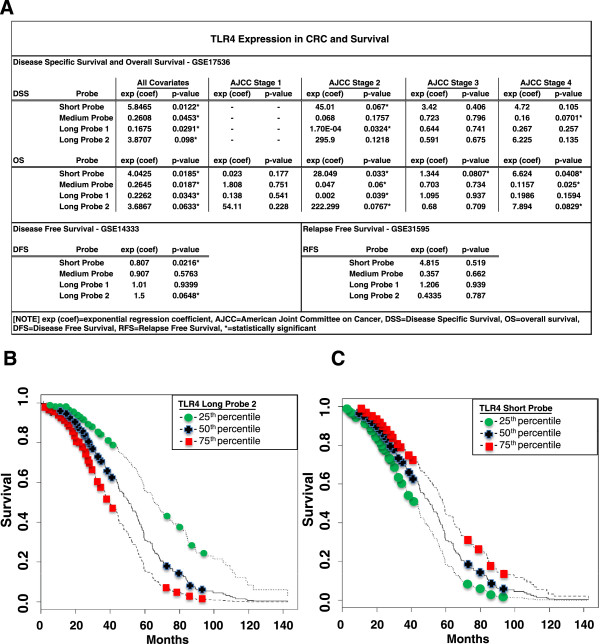
**CRC Survival and Relationship to TLR4 Expression. A)** DSS, OS, DFS, and RFS are shown with their associated exponential regression coefficients (exp (coef)) and significance levels. Note that the direction of the coefficients varied depending on probe. **B** and **C)** Cox Proportional Hazard Curves (GSE14333) for DFS based on level of TLR4 expression. Three curves are generated for each probe based on quartiles of TLR4 expression. These graphs demonstrate that probability of DFS is significantly associated with TLR4 expression, and the direction of the association is probe-dependent. **B)** For TLR4 long probe 2,DFS is lowest in the group with the highest level of expression (75^th^ percentile). Cut-off values for TLR4 expression were as follows: 5.0 (25^th^ percentile), 6.8 (50^th^ percentile), and 8.0 (75^th^ percentile). **C)** For the TLR4 Short Probe, higher levels of expression result in improved DFS. The same cut-off values were used as in Figure 3B.

In a large series of 232 CRCs, TLR4 expression was related to OS when all stages of CRC were considered in aggregate (Figure 3A) (GSE17536) [25]. Specifically, TLR4 was significantly associated with both DSS and OS in AJCC stages 2 and 4. Across all stages, we found that for two of the TLR4 probes (Short and Long2) a higher expression correlated with improved OS (exp(coef)_short_ = 4.04, p = 0.019; exp(coef)_long2_ = 3.69, p = 0.06). By contrast, the remaining probes (Medium and Long1) showed decreased expression with improved survival (exp(coef)_medium_ = 0.26, p = 0.019; exp(coef)_long1_ = 0.22, p = 0.034).

The association between survival and TLR4 expression was corroborated by a strong correlation between TLR4 expression (Short and Long2) and DFS among 290 colon cancer patients ranging from Duke’s stages A through D (exp (coef) 0.78, p = 0.0008 and exp (coef) 1.47, p = 0.0006) (GSE14333) [17]. TLR4 expression levels were divided into quartiles by probe. Survival curves were constructed per probe, meant to represent low, average, and high expression (Figure 3B, 3C). For Long2, higher expression of TLR4 was associated with lower probability of DFS (Figure 3B). The inverse relationship was demonstrated for Short (Figure 3C). This association between DFS and TLR4 expression was not supported by other GSE series examining the endpoints of OS, DFS (GSE12945) [26], relapse-free survival (GSE8671) [18] and recurrence-free survival (GSE33113) [27]. In a separate series of 48 sporadic colon cancer samples, no association between TLR4 expression and survival was observed (exp (coef) = 1.13, p = 0.61) (GSE16125) [28].

When differentiating colon from rectal cancers, the tumor location was not significant in any models of survival, p > 0.80.

#### TLR4 expression is strongly associated with recurrence after chemotherapy

Among 100 stage 3 colon cancers, significant associations between TLR4 expression and tumor recurrence were observed [29] with higher expression for all four probes among patients with recurrence compared to those without (p-values = 0.036, 0.076, 0.087, and 0.056 for probes Short, Probe, Long1, and Long2, respectively) (GSE5206). These data suggest that specific TLR4 transcripts may be incorporated in predictive models of colon cancer survival and recurrence.

#### Lower TLR4 expression and microsatellite instability (MSI)

Microsatellite unstable tumors are associated with defects in mismatch repair but have improved prognosis [30]. We investigated the relationship between MSI and TLR4 expression. Among 77 microsatellite stable (MSS) and 78 MSI colon cancers, TLR4 expression strongly correlated with MSI status, with MSI tumors having significantly lower TLR4 expression than MSS comparators (GSE13294) [31].MSI was associated with lower expression of TLR4.

### IHC staining for TLR4

**Figure 4:**
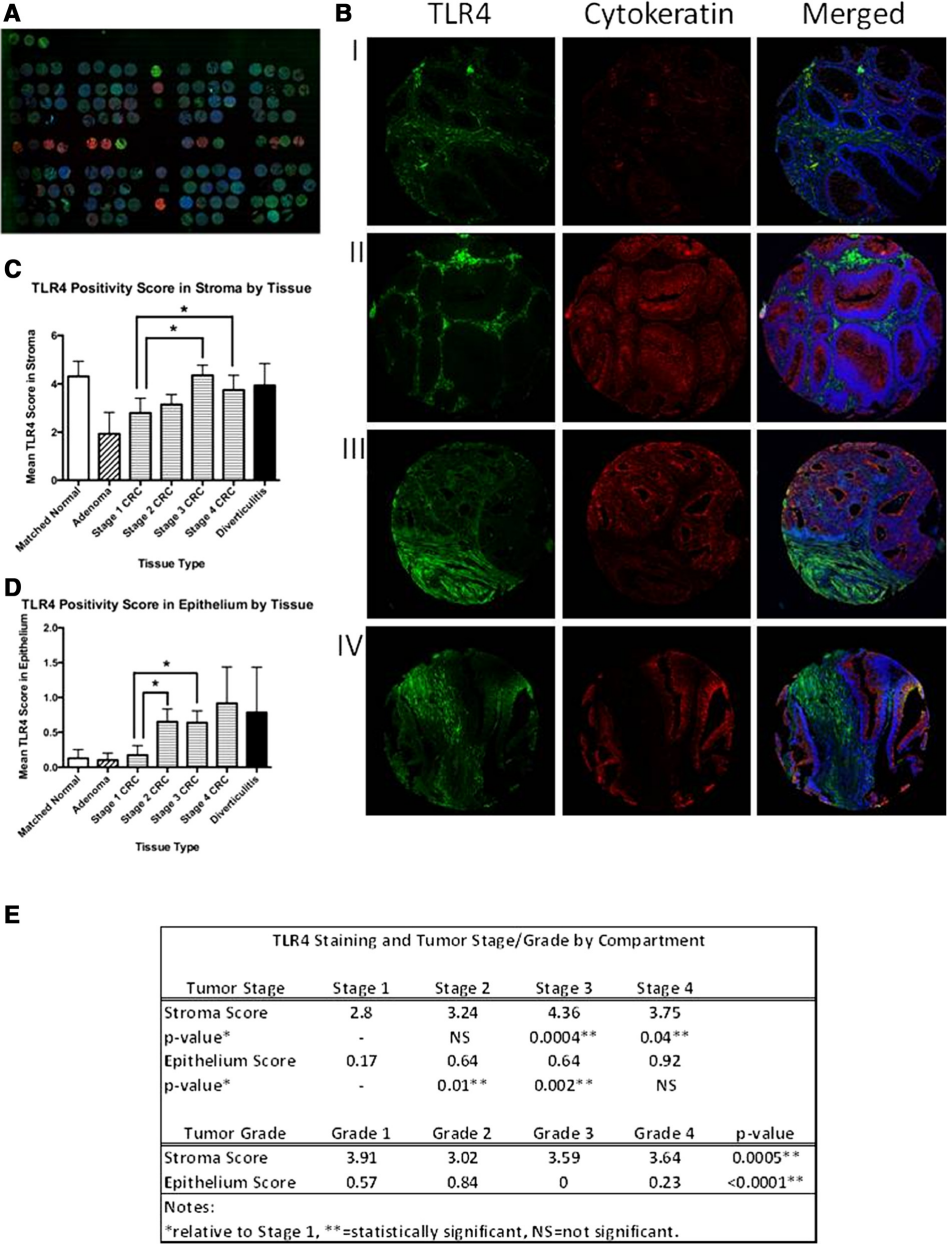
**Immunofluorescent staining of TMAs. A)** Low power (10x) view of NCI TMA slide stained for TLR4 (green), intestinal epithelium/pan-cytokeratin (red), and nucleus/DAPI (blue). **B)** Representative tissue cores from normal (I), adenomatous polyps (II), and CRC (III and IV) are shown. **C** and **D)** TLR4 staining score by tissue type and tissue compartment (stroma vs epithelium) are shown. **C)** TLR4 staining in the tumor stroma had a significantly higher average intensity score for stages 3 and 4 CRC when compared to stage 1. **D)** TLR4 staining in the tumor epithelium had a significantly higher average intensity score for stages 2 and 3 when compared to stage 1. **E)** TLR4 staining by compartment broken down by stage (controlling for grade) and grade (controlling for stage).

The data from bioinformatics analysis of expression arrays demonstrate an increase in TLR4 genomic expression in neoplastic colon tissue, with relatively high expression in the stromal compartment in particular. We next wished to examine whether this could be seen at the protein level using NCI TMAs. TMAs consisting of 182 independent cancers, 19 adenomas, and matched normal tissue were examined for TLR4 expression (Figure 4A,4B) [11]. Select cores were excluded from the analysis due to poor sample integrity or staining quality (18 out of 239). The stroma of 82/174 (47.1%) of CRCs was positive for TLR4 expression (score > 3). 62/174 (35.6%) of tumor stroma were strongly positive (score > 5). The epithelium of 11/174 (6.32%) of CRCs was positive for TLR4 expression; 6/174 (3.45%) of tumor epithelia were strongly positive.

#### TLR4 staining in the tumor stroma and epithelium increases with tumor stage

Using semi-quantitative scoring, a positive relationship was noted between TLR4 staining score in the tumor stroma and tumor stage, controlling for histology grade, with significantly higher intensity score for stages 3 and 4 compared to stage 1 (Stage 1 = 2.80, Stage 2 = 3.24, Stage 3 = 4.36, Stage 4 = 3.75; p = NS, 0.0004, and 0.04, respectively) (Figure 4C,4D,4E). A positive relationship was noted between TLR4 staining score in the tumor epithelium and tumor stage, controlling for histology grade, with significantly higher intensity score for stages 2 and 3 compared to stage 1 (Stage 1 = 0.17, Stage 2 = 0.64, Stage 3 = 0.64, Stage 4 = 0.92; p =0.01, 0.002, and NS, respectively). These data suggest that TLR4 protein expression mirrors what we found in the transcriptome data.

#### Tumor stroma, epithelium, and grade

TLR4 staining scores were recorded in the tumor stroma and stratified by tumor grade as follows: well-differentiated = 3.91, moderately-differentiated = 3.02, poorly-differentiated = 3.59, undifferentiated = 3.64 (ANOVA comparing all four categories, p = 0.0005). The TLR4 staining score in the tumor epithelium was classified by tumor grade: well-differentiated = 0.57, moderately-differentiated = 0.84, poorly-differentiated = 0.00, or undifferentiated = 0.23 (ANOVA comparing all four categories, p = 9.99 × 10^−9^). Well-differentiated tumors had a higher stroma:epithelium TLR4 staining ratio than moderately-differentiated tumors (6.86 vs 3.59, respectively). Poor- and un-differentiated tumors had modest stromal staining but little to absent epithelial staining.

#### Survival and recurrence

A trend toward statistical significance was observed between increased TLR4 stromal staining and decreased OS (p = 0.16) after correcting for both stage and grade. Marginal significance was observed for the relationship describing increased epithelial TLR4 staining and decreased OS (p = 0.11). No relation between TLR4 expression and time to tumor recurrence was noted.

#### TLR4 staining in polyps

Given the small number of interpretable adenomatous tissue cores on the NCI TMA (n = 15), an additional TMA with adenomas and normal controls was stained. Small sample sizes prevented achievement of significance for all endpoints. Mean TLR4 stromal staining scores were lower in adenomatous polyps (n = 14) than normal tissue (n = 12) controls (adenoma 2.29 versus normal 3.5, W = 95, p = 0.58). Mean TLR4 epithelial staining scores were lower in adenomatous polyps than normal tissue controls (adenoma 0.57 versus normal 0.67, W = 67, p = 0.30). Mean TLR4 stromal and epithelial staining scores among inflammatory polyps (IP) were higher than normal tissue controls (stroma: IP 5.6 vs normal 3.5, p = 0.22 and epithelium: IP 1.8 versus normal 0.67, p = 0.81). These under-powered observations support the expected finding that inflamed polyps would manifest higher TLR4 levels.

#### Increased TLR4 expression in the epithelium and pericryptal myofibroblasts (PCMs) in CRCs

**Figure 5:**
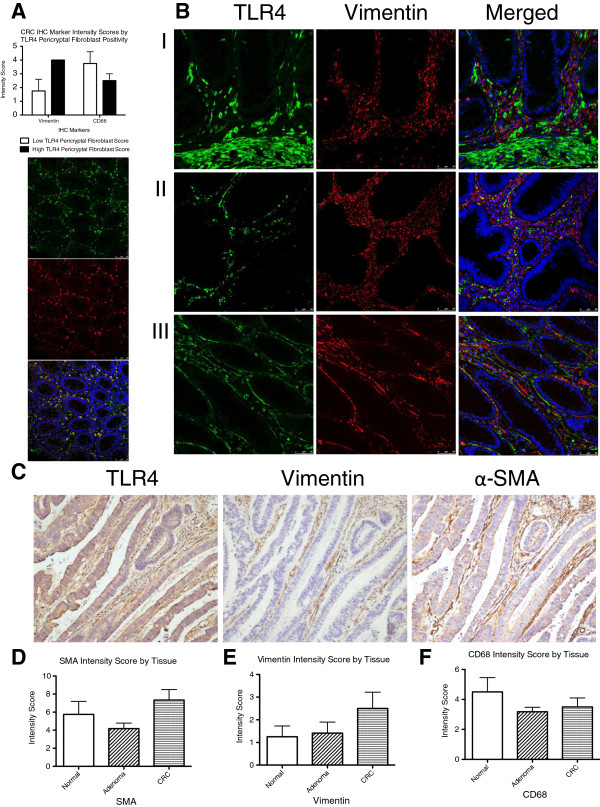
**Pericryptal Myofibroblasts are Responsible for Increased TLR4 Expression in a Subset of CRCs. A)** CRCs were separated into two groups representing low- and high- stromal expression of TLR4 by IHC staining. In normal tissue, stromal TLR4 expression is mainly due to macrophages (Green: TLR4, Red: CD68, Merge: TLR4 + CD68 + DAPI (blue)). Conversely, in CRCs increased vimentin and decreased CD68 staining in the pericryptal space confirm that this signal was due to pericryptal myofibroblasts and not related to tumor-associated macrophages. **B)** Double-stained immunofluorescence for TLR4 (green) and vimentin (red) in normal (I), adenoma (II), and colon adenocarcinoma (III) (10×). In the stromal compartment of CRCs, immunofluorescent staining for TLR4 localized to the pericryptal myofibroblasts in a subset of samples. **C)** IHC staining of colon adenocarcinoma for TLR4, vimentin, and α-SMA (40×). Staining co-localizes to the pericryptal space, confirming the signal arises from pericryptal myofibroblasts. **D**, **E**, and **F)** An increase in IHC staining for α-SMA and vimentin was noted in CRCs when compared to normal or low grade dysplasia. A decrease in staining for CD68 positive macrophages was observed with higher degrees of dysplasia.

Using cytokeratin staining to identify epithelium, we found that TLR4 is over-expressed in a subset of tumors and that the expression increases from normal to adenoma to cancer. We also observed increased TLR4 staining in the cytokeratin-negative stroma. Given the increased stromal staining of TLR4, we wished to clarify which cell types comprise the TLR4-positive stroma in CRCs. Clinical insights from hematoxylin sections suggested fibroblasts as the source for this increased intensity. IHC staining of unmatched normal colon, adenoma, and CRC patient samples for TLR4, vimentin, and CD68 was performed in a limited cohort to identify myofibroblasts and macrophages, respectively (Figure 5). In the stromal compartment of a subset of CRCs, IHC staining for TLR4 localized to the PCMs. Vimentin and CD68 staining in the stromal compartments of CRCs with low and high expression of TLR4 confirmed that the increased TLR4 signal was localized to PCMs and not related to tumor-associated macrophages.

## Discussion

We have leveraged available transcriptome databases and well-designed TMAs to address the biologic role of TLR4 in colon dysplasia. The current work both confirms hypotheses engendered from our basic science work and generates new hypotheses about TLR4 signaling and sporadic CRC. In our animal models, we have found that mice constitutively expressing TLR4 have an increased severity of chemically-induced colitis and develop more colonic tumors [8]. This tumor burden could be attenuated using TLR4-inhibiting antibody. Bringing relevance to humans with colitis-associated cancers (CACs), TLR4 is over-expressed in the majority, with increasing expression with dysplastic progression [8]. We have further shown that TLR4 leads to activation of the Wnt/β-catenin pathway which is activated in most sporadic CRCs [9]. Analogous to CACs, we have found an association between TLR4 expression in sporadic CRC and the progression of neoplasia, stage, DFS, and MSS. In particular, an increased expression of TLR4 in the tumor stroma relative to the malignant epithelium was noted. These expression data were validated with IHC showing a similar stroma:epithelium gradient. 35.6% of CRCs demonstrate high levels of TLR4 protein in the tumor stroma, while 3.45% have high levels in the tumor epithelium. Within the stroma, high TLR4 signal localized to the PCMs in particular.

Others using different methodology and smaller numbers demonstrated that TLR4 is associated with tumor stage. Cammarota, et al. have previously reported that stromal TLR4 expression in CRCs is associated with disease progression [13]. In this series, CRC relapse was predicted by increased stromal TLR4 for stage pT3, lending credence to the predictive capability of this marker [13]. Our study corroborated these findings using a larger sample of tissues, and answered the subsequent question of whether TLR4 transcripts can be associated with additional CRC endpoints. We confirmed that TLR4 transcript levels were related to colonic dysplasia, CRC stage, and survival.

In a separate series, Wang, et al. demonstrated high TLR4 expression in 20% of CRCs by immunostaining and its association with shorter OS. Both the expression of TLR4 and its co-receptor MyD88 were associated with the presence of liver metastases [12]. In xenograft models of CRC, TLR4 silencing with RNA interference decreases the metastatic tumor burden in the liver [32]. Proliferation of TLR4-expressing breast tumors has also been stunted with TLR4-inhibition *in vitro*[33]. In contrast, data from unrelated CRC cell line populations support the loss of expression or down-regulation of TLR4 in metastases compared to earlier stage tumors [34]. The conflicting observations with respect to TLR4’s role in CRC metastases likely is a reflection of the biologic variation in CRCs, with TLR4 being over-expressed in a subset. Our study did not find a clear association with metastases.

Our study used IF and IHC to understand the location of TLR4 expression in colonic neoplasia. In agreement with Cammarota and Wang, we found that TLR4 protein expression in the stromal compartment was associated with more advanced stages of colon cancer. But we also found that normal stroma has TLR4 positive cells, largely CD68+ macrophages. Our transcriptome data demonstrated high TLR4 expression in adenomas relative to normal tissue and, to a lesser degree, higher expression relative to cancer. We speculate that adenomas may represent a more homogeneous tissue than cancer or that TLR4 plays an important role in tumor promotion from adenoma to cancer.

Our study and Cammarota found that stromal TLR4 expression is associated with cancer outcomes. In addition to the previous documentation of TLR4 expression by the submucosal vascular endothelium or hematopoietic mononuclear cells, our study demonstrated that PCMs also contribute to the TLR4 expression found in the stroma [13]. These PCMs have previously been recognized as a discrete cell type in colonic adenomas, displaying a unique pattern of surface markers [35,36]. Increased density of these fibroblasts has been described in the stroma of digestive tract neoplasia [37]. They may originate from deeper layers of the intestine, and have been proposed as tumor propagators via the epithelial-to-stromal transition [38,39]. We propose that TLR4 on the surface of these PCMs may play a role in the interaction between the epithelial and stromal compartments to promote neoplasia. These myofibroblasts have been shown *in vitro* to respond to TLR signals and may therefore contribute to tumor promotion by secreting trophic factors in response to bacterial ligands [40].

One of the interesting findings among the platforms containing multiple TLR4 probes was a marked divergence of transcripts with clinical outcomes. In particular, the direction and magnitude of specific TLR4 transcript expression on survival was evident, where TLR4 probes fall into two distinct groups, each of which targets a different transcript variant. There exist four recognized mRNA TLR4 products (Figure 1B) [41]. Four probes from the commercial platform correspond to longer transcripts, while the remaining two probes are associated specifically with shorter mRNAs. The dichotomous relationship between RNA transcripts and clinical outcomes raises the possibility that different TLR4 transcripts or their relative ratios have different biological activities and consequences. The immunology literature supports the notion that alternative splicing of genes involved in innate immunity regulates their function [42-44]. In particular, alternative splicing has been observed in TLR family members expressed in response to LPS [43]. This splicing phenomenon may explain the opposing survival results observed herein. Epigenetic events, like hypermethylation of gene promoters which occur frequently in CRCs, may also play a role in the expression of varying transcripts [45]. Other post-transcriptional regulatory events may also contribute; trafficking of transcripts by microRNAs offers another plausible explanation. miR21, a microRNA present in many tumors, also has been shown to down-regulate TLR4 [46]. We speculate that the type of TLR4 mRNA/protein product regulates biological events, as may non-coding TLR4 transcripts found in genome browsers (Figure 1C). Bench and animal experiments are required to interrogate the mechanism for the functional differences in TLR4 transcripts.

The authors acknowledge the limitations of this study. Most notably, the TMA histologic scoring was based on cores; accordingly, TLR4 positivity may have been underestimated given the heterogeneous nature of CRCs and sampling error inherent in cores. We did not incubate TMA controls with only secondary antibody (TLR4) without the primary antibody; our controls consisted of unmatched, uninvolved colonic tissue. Finally, RNA expression and protein staining conclusions were drawn from unmatched samples in some instances.

## Conclusions

TLR4 may play distinct roles in the transition from normal colon to adenoma and from a local to a more advanced tumor. In our animal models, the absence of TLR4 protects against developing dysplasia. In animals with colonic tumors, treatment with an anti-TLR4 antibody results in smaller tumors. Our animal data combined with the human data presented in the current manuscript argue for studying TLR4 antagonists in cancer. These findings also support further investigation of TLR4 in predictive models of colon cancer outcomes.

## Abbreviations

CRC: Colorectal cancer; TLR: Toll-like receptor; DAMP: Damage-associated molecular pattern; LPS: Lipopolysaccharide; IHC: Immunohistochemical; IF: Immunofluorescent; TMA: Tissue microarray; GEO: Gene expression omnibus; OS: Overall survival; DSS: Disease specific survival; DFS: Disease free; MSI: Microsatellite instability; CDP: Cancer diagnosis program; AJCC: American joint committee on cancer; FC: Fold change; MSS: Microsatellite stable; PCM: Peri-cryptal myofibroblast; CAC: Colitis-associated cancer.

## Competing interests

The authors do not have any relevant financial interests related to the work described in this manuscript.

## Authors’ contributions

DAS participated in the design of the study, acquired the data, interpreted the data, and drafted the manuscript. RS performed the immunofluorescent and immunohistochemical staining. PAB participated in the interpretation and scoring of immunofluorescence. MTG participated in the interpretation and scoring of immunofluorescence. MTP participated in the interpretation and scoring of immunohistochemical stains. MTA participated in the design of the study and interpretation of results. JC participated in the design of the study, performed the statistical analysis, and interpreted results. All authors participated in the preparation of the manuscript as well as reviewed and approved the final manuscript.

## References

[B1] Terzic J Grivennikov S Karin E Karin M Inflammation and colon cancer Gastroenterology 2010 138 6 2101 2114 e2105 10.1053/j.gastro.2010.01.058 20420949

[B2] Eckburg PB Bik EM Bernstein CN Purdom E Dethlefsen L Sargent M Gill SR Nelson KE Relman DA Diversity of the human intestinal microbial flora Science 2005 308 5728 1635 1638 10.1126/science.1110591 15831718 PMC1395357

[B3] Wells JM Rossi O Meijerink M van Baarlen P Epithelial crosstalk at the microbiota-mucosal interface Proc Natl Acad Sci U S A 2011 108 Suppl 1 4607 4614 20826446 10.1073/pnas.1000092107 PMC3063605

[B4] Poxton IR Brown R Sawyerr A Ferguson A The mucosal anaerobic gram-negative bacteria of the human colon Clin Infect Dis 1997 25 Suppl 2 S111 S113 9310645 10.1086/516189

[B5] Zheng L Riehl TE Stenson WF Regulation of colonic epithelial repair in mice by Toll-like receptors and hyaluronic acid Gastroenterology 2009 137 6 2041 2051 10.1053/j.gastro.2009.08.055 19732774 PMC2789856

[B6] Ehrchen JM Sunderkotter C Foell D Vogl T Roth J The endogenous Toll-like receptor 4 agonist S100A8/S100A9 (calprotectin) as innate amplifier of infection, autoimmunity, and cancer J Leukoc Biol 2009 86 3 557 566 10.1189/jlb.1008647 19451397

[B7] Fukata M Chen A Vamadevan AS Cohen J Breglio K Krishnareddy S Hsu D Xu R Harpaz N Dannenberg AJ Subbaramaiah K Cooper HS Itzkowitz SH Abreu MT Toll-like receptor-4 promotes the development of colitis-associated colorectal tumors Gastroenterology 2007 133 6 1869 1881 10.1053/j.gastro.2007.09.008 18054559 PMC2180834

[B8] Fukata M Shang L Santaolalla R Sotolongo J Pastorini C España C Ungaro R Harpaz N Cooper HS Elson G Kosco-Vilbois M Zaias J Perez MT Mayer L Vamadevan AS Lira SA Abreu MT Constitutive activation of epithelial TLR4 augments inflammatory responses to mucosal injury and drives colitis-associated tumorigenesis Inflamm Bowel Dis 2011 17 7 1464 1473 10.1002/ibd.21527 21674704 PMC3117047

[B9] Santaolalla R Sussman DA Ruiz JR Davies JM Pastorini C España CL Sotolongo J Burlingame O Bejarano PA Philip S Ahmed MM Ko J Dirisina R Barrett TA Shang L Lira SA Fukata M Abreu MT TLR4 activates the beta-catenin pathway to cause intestinal neoplasia PLoS ONE 2013 8 5 e63298 10.1371/journal.pone.0063298 23691015 PMC3653932

[B10] Gene expression omnibus http://www.ncbi.nlm.nih.gov/geo/

[B11] Colon cancer tissue microarray case sets http://dctd.cancer.gov/ProgramPages/cdp/tools_disease-specific.htm

[B12] Wang EL Qian ZR Nakasono M Tanahashi T Yoshimoto K Bando Y Kudo E Shimada M Sano T High expression of Toll-like receptor 4/myeloid differentiation factor 88 signals correlates with poor prognosis in colorectal cancer Br J Cancer 2010 102 5 908 915 10.1038/sj.bjc.6605558 20145615 PMC2833250

[B13] Cammarota R Bertolini V Pennesi G Bucci EO Gottardi O Garlanda C Laghi L Barberis MC Sessa F Noonan DM Albini A The tumor microenvironment of colorectal cancer: stromal TLR-4 expression as a potential prognostic marker J Transl Med 2010 8 112 10.1186/1479-5876-8-112 21059221 PMC2997091

[B14] RDCTeam R: A Language and Environment for Statistical Computing 2010 Vienna, Austria: R Foundation for Statistical Computing

[B15] Benjamini Y Hochberg Y Controlling the false discovery rate: a practical and powerful approach to multiple testing J Roy Stat Soc B Met 1995 57 1 289 300

[B16] Troyanskaya OG Garber ME Brown PO Botstein D Altman RB Nonparametric methods for identifying differentially expressed genes in microarray data Bioinformatics (Oxford, England) 2002 18 11 1454 1461 10.1093/bioinformatics/18.11.1454 12424116

[B17] Jorissen RN Gibbs P Christie M Prakash S Lipton L Desai J Kerr D Aaltonen LA Arango D Kruhøffer M Orntoft TF Andersen CL Gruidl M Kamath VP Eschrich S Yeatman TJ Sieber OM Metastasis-associated gene expression changes predict poor outcomes in patients with Dukes stage B and C colorectal cancer Clin Cancer Res 2009 15 24 7642 7651 10.1158/1078-0432.CCR-09-1431 19996206 PMC2920750

[B18] Sabates-Bellver J Van der Flier LG de Palo M Cattaneo E Maake C Rehrauer H Laczko E Kurowski MA Bujnicki JM Menigatti M Luz J Ranalli TV Gomes V Pastorelli A Faggiani R Anti M Jiricny J Clevers H Marra G Transcriptome profile of human colorectal adenomas Mol Cancer Res 2007 5 12 1263 1275 10.1158/1541-7786.MCR-07-0267 18171984

[B19] Skrzypczak M Goryca K Rubel T Paziewska A Mikula M Jarosz D Pachlewski J Oledzki J Ostrowski J Modeling oncogenic signaling in colon tumors by multidirectional analyses of microarray data directed for maximization of analytical reliability PLoS ONE 2010 5 10 10.1371/journal.pone.0013091 PMC2948500 20957034

[B20] Lips EH van Eijk R de Graaf EJ Oosting J de Miranda NF Karsten T van de Velde CJ Eilers PH Tollenaar RA van Wezel T Morreau H Integrating chromosomal aberrations and gene expression profiles to dissect rectal tumorigenesis BMC Cancer 2008 8 314 10.1186/1471-2407-8-314 18959792 PMC2584339

[B21] Nishida N Nagahara M Sato T Mimori K Sudo T Tanaka F Shibata K Ishii H Sugihara K Doki Y Mori M Microarray analysis of colorectal cancer stromal tissue reveals upregulation of two oncogenic miRNA clusters Clin Cancer Res 2012 18 11 3054 3070 10.1158/1078-0432.CCR-11-1078 22452939

[B22] Gray R Barnwell J McConkey C Hills RK Williams NS Kerr DJ Adjuvant chemotherapy versus observation in patients with colorectal cancer: a randomised study Lancet 2007 370 9604 2020 2029 10.1016/S0140-6736(07)61866-2 18083404

[B23] André T Boni C Mounedji-Boudiaf L Navarro M Tabernero J Hickish T Topham C Zaninelli M Clingan P Bridgewater J Tabah-Fisch I de Gramont A Oxaliplatin, fluorouracil, and leucovorin as adjuvant treatment for colon cancer N Engl J Med 2004 350 23 2343 2351 10.1056/NEJMoa032709 15175436

[B24] Thorsteinsson MKL Lund LR Sørensen LT Gerds TA Jess P Olsen J Gene expression profiles in stage II and III colon cancer. Application of a 128-gene signature Int J Colorectal Dis 2012 27 12 1579 1586 10.1007/s00384-012-1517-4 22710688

[B25] Smith JJ Deane NG Wu F Merchant NB Zhang B Jiang A Lu P Johnson JC Schmidt C Bailey CE Eschrich S Kis C Levy S Washington MK Heslin MJ Coffey RJ Yeatman TJ Shyr Y Beauchamp RD Experimentally derived metastasis gene expression profile predicts recurrence and death in patients with colon cancer Gastroenterology 2010 138 3 958 968 10.1053/j.gastro.2009.11.005 19914252 PMC3388775

[B26] Staub E Groene J Heinze M Mennerich D Roepcke S Klaman I Hinzmann B Castanos-Velez E Pilarsky C Mann B Brümmendorf T Weber B Buhr HJ Rosenthal A An expression module of WIPF1-coexpressed genes identifies patients with favorable prognosis in three tumor types J Mol Med (Berl) 2009 87 6 633 644 10.1007/s00109-009-0467-y 19399471 PMC2688022

[B27] de Sousa E Melo F Colak S Buikhuisen J Koster J Cameron K de Jong JH Tuynman JB Prasetyanti PR Fessler E van den Bergh SP Rodermond H Dekker E van der Loos CM Pals ST van de Vijver MJ Versteeg R Richel DJ Vermeulen L Medema JP Methylation of cancer-stem-cell-associated Wnt target genes predicts poor prognosis in colorectal cancer patients Cell Stem Cell 2011 9 5 476 485 10.1016/j.stem.2011.10.008 22056143

[B28] Reid JF Gariboldi M Sokolova V Capobianco P Lampis A Perrone F Signoroni S Costa A Leo E Pilotti S Pierotti MA Integrative approach for prioritizing cancer genes in sporadic colon cancer Genes Chromosomes Cancer 2009 48 11 953 962 10.1002/gcc.20697 19672874

[B29] Kaiser S Park YK Franklin JL Halberg RB Yu M Jessen WJ Freudenberg J Chen X Haigis K Jegga AG Kong S Sakthivel B Xu H Reichling T Azhar M Boivin GP Roberts RB Bissahoyo AC Gonzales F Bloom GC Eschrich S Carter SL Aronow JE Kleimeyer J Kleimeyer M Ramaswamy V Settle SH Boone B Levy S Graff JM Transcriptional recapitulation and subversion of embryonic colon development by mouse colon tumor models and human colon cancer Genome Biol 2007 8 7 R131 10.1186/gb-2007-8-7-r131 17615082 PMC2323222

[B30] Gryfe R Kim H Hsieh ET Aronson MD Holowaty EJ Bull SB Redston M Gallinger S Tumor microsatellite instability and clinical outcome in young patients with colorectal cancer N Engl J Med 2000 342 2 69 77 10.1056/NEJM200001133420201 10631274

[B31] Jorissen RN Lipton L Gibbs P Chapman M Desai J Jones IT Yeatman TJ East P Tomlinson IP Verspaget HW Aaltonen LA Kruhøffer M Orntoft TF Andersen CL Sieber OM DNA copy-number alterations underlie gene expression differences between microsatellite stable and unstable colorectal cancers Clin Cancer Res 2008 14 24 8061 8069 10.1158/1078-0432.CCR-08-1431 19088021 PMC2605660

[B32] Earl TM Nicoud IB Pierce JM Wright JP Majoras NE Rubin JE Pierre KP Gorden DL Chari RS Silencing of TLR4 decreases liver tumor burden in a murine model of colorectal metastasis and hepatic steatosis Ann Surg Oncol 2009 16 4 1043 1050 10.1245/s10434-009-0325-8 19165543

[B33] Yang H Zhou H Feng P Zhou X Wen H Xie X Shen H Zhu X Reduced expression of Toll-like receptor 4 inhibits human breast cancer cells proliferation and inflammatory cytokines secretion J Exp Clin Cancer Res 2010 29 92 10.1186/1756-9966-29-92 20618976 PMC2913950

[B34] Simiantonaki N Kurzik-Dumke U Karyofylli G Jayasinghe C Michel-Schmidt R Kirkpatrick CJ Reduced expression of TLR4 is associated with the metastatic status of human colorectal cancer Int J Mol Med 2007 20 1 21 29 17549384

[B35] Adegboyega PA Mifflin RC DiMari JF Saada JI Powell DW Immunohistochemical study of myofibroblasts in normal colonic mucosa, hyperplastic polyps, and adenomatous colorectal polyps Arch Pathol Lab Med 2002 126 7 829 836 12088453 10.5858/2002-126-0829-ISOMIN

[B36] Adegboyega PA Ololade O Saada J Mifflin R di Mari JF Powell DW Subepithelial myofibroblasts express cyclooxygenase-2 in colorectal tubular adenomas Clin Cancer Res 2004 10 17 5870 5879 10.1158/1078-0432.CCR-0431-03 15355919

[B37] Kalluri R Zeisberg M Fibroblasts in cancer Nat Rev Cancer 2006 6 5 392 401 10.1038/nrc1877 16572188

[B38] Ban S Mitsuhashi T Shimizu M Immunohistochemical study of myofibroblasts in colorectal epithelial lesions Arch Pathol Lab Med 2003 127 12 1551 1553 author reply 1551–1552 14655255 10.5858/2003-127-924-AROAFL

[B39] Worthley DL Giraud AS Wang TC Stromal fibroblasts in digestive cancer Cancer Microenviron 2010 3 1 117 125 10.1007/s12307-009-0033-8 21209778 PMC2970811

[B40] Otte JM Rosenberg IM Podolsky DK Intestinal myofibroblasts in innate immune responses of the intestine Gastroenterology 2003 124 7 1866 1878 10.1016/S0016-5085(03)00403-7 12806620

[B41] University of California Santa Clara genome browser http://genome.ucsc.edu/cgi-bin/hgTracks?hgHubConnect.destUrl=..%2Fcgi-bin%2FhgTracks&clade=mammal&org=Human&db=hg19&position=chr9%3A120466460-120479766&hgt.suggest=tlr4&hgt.suggestTrack=knownGene&Submit=submit&hgsid=279196009&knownGene=pack

[B42] Lynch KW Consequences of regulated pre-mRNA splicing in the immune system Nat Rev Immunol 2004 4 12 931 940 10.1038/nri1497 15573128

[B43] Wells CA Chalk AM Forrest A Taylor D Waddell N Schroder K Himes SR Faulkner G Lo S Kasukawa T Kawaji H Kai C Kawai J Katayama S Carninci P Hayashizaki Y Hume DA Grimmond SM Alternate transcription of the Toll-like receptor signaling cascade Genome Biol 2006 7 2 R10 10.1186/gb-2006-7-2-r10 16507160 PMC1431733

[B44] Grigoryev YA Kurian SM Nakorchevskiy AA Burke JP Campbell D Head SR Deng J Kantor AB Yates JR 3rd Salomon DR Genome-wide analysis of immune activation in human T and B cells reveals distinct classes of alternatively spliced genes PLoS ONE 2009 4 11 e7906 10.1371/journal.pone.0007906 19936255 PMC2775942

[B45] Moon JW Lee SK Lee JO Kim N Lee YW Kim SJ Kang HJ Kim J Kim HS Park SH Identification of novel hypermethylated genes and demethylating effect of vincristine in colorectal cancer J Exp Clin Cancer Res 2014 33 4 10.1186/1756-9966-33-4 24393480 PMC3923411

[B46] Sheedy FJ Palsson-McDermott E Hennessy EJ Martin C O’Leary JJ Ruan Q Johnson DS Chen Y O’Neill LA Negative regulation of TLR4 via targeting of the proinflammatory tumor suppressor PDCD4 by the microRNA miR-21 Nat Immunol 2010 11 2 141 147 10.1038/ni.1828 19946272

